# Anti-N-Methyl-D-Aspartate Receptor Encephalitis: Neuropsychiatric and Multidisciplinary Approach to a Patient Not Responding to First-Line Treatment

**DOI:** 10.7759/cureus.25751

**Published:** 2022-06-08

**Authors:** Arsen S Askandaryan, Abbas Naqvi, Amanda Varughese, Dina Rimawi

**Affiliations:** 1 Psychiatry, Jamaica Hospital Medical Center, New York, USA

**Keywords:** cns manifestations, second line drugs, autoimmune flare-up, anti-nmda receptor encephalitis, nmda receptor antibodies, autoimmune encephalitis

## Abstract

The understanding of anti-NMDA (N-methyl-D-aspartate) receptor encephalitis, recognized by Dalmau and colleagues in 2007, has come a long way in helping clinicians to recognize the significance of rapidly progressive psychiatric symptoms in patients who are actually suffering from autoimmune disease. This subtype of autoimmune encephalitis manifests from antibodies that target the NR1 and/or NR2 subunits of NMDA receptors in serum or cerebrospinal fluid. Since gaining notoriety among neurologists, it has shown an etiologic predilection for children, adolescents, and young adult females, often associated with ovarian teratomas. Conversely, it affects young males as well, though it is rarer to find co-occurring tumors. It is a multistage disorder, initially presenting with psychiatric symptoms that progress in varying fashion, including headache, fever, nuchal rigidity, emesis, seizure, autonomic instability, auditory and visual hallucinations, delusional ideation, agitation, altered sensorium, and motor disturbances (i.e. dyskinesia, catatonia, etc.). Early diagnosis is critical due to the relatively high (25%) mortality rate. In this case, we present the case of a 30-year-old male who presented to our institution’s Comprehensive Psychiatric Emergency Program (CPEP) exhibiting bizarre behavior and visual hallucinations, and was later confirmed to have anti-NMDA receptor encephalitis. The case report highlights the risk factors, disease course, and treatment modalities of anti-NMDA receptor encephalitis with special emphasis on the subsect of patients who may not respond to first-line therapies.

## Introduction

Of central importance is recognizing anti-NMDA (N-methyl-D-aspartate) receptor encephalitis clinically to provide patients with prompt and appropriate treatment. From a psychiatric perspective, it is not uncommon to encounter these patients in acute emergency settings (i.e. Comprehensive Psychiatric Emergency Program [CPEP], medical ER, etc.). The nature of presenting signs and symptoms may mimic psychotic features, often mislabeled as manifestations of underlying psychiatric and substance use pathologies. It is imperative that clinicians familiarize themselves with the nuances of the disease to accurately and efficiently decide on a differential diagnosis, and to parse through what tests and levels of care are appropriate for the patient. Perhaps one of the most important, yet understated, goals is the emphasis on interdisciplinary communication. In this patient’s case of NMDA receptor encephalitis, four separate teams were involved in his care before discharge, and each played a unique, integral role in managing the patient effectively, making effective communication a necessity for a positive outcome [1-4].

## Case presentation

The patient of interest is a 30-year-old Hispanic male with no significant psychiatric history, and past medical history of crack cocaine, nicotine, and alcohol use, and traumatic brain injury one year earlier after an altercation where he was hit in the head with a bat. Although, the patient's urine toxicology was negative on admission, and collateral information gathered showed only that he was a daily cigarette smoker with an unknown quantity and he drank mainly at social gatherings with family. There was one mention of cocaine use, but this was remote, noted as an isolated event 1-2 years prior to this presentation. With regard to the head injury, the patient suffered a blunt trauma, described by the family as the patient being involved in an altercation where he was hit with a bat, but there are no medical records to provide details, and the family was unable to provide further information on that. The patient was brought into the CPEP by ambulance, activated by family, due to worsening confusion and bizarre behavior.

One week earlier, he was evaluated by another facility’s ER nurse practitioner after complaining of flu-like symptoms and frontal headache for three weeks. He was diagnosed with acute sinusitis and discharged with ibuprofen and Augmentin for a seven-day course. On the day of the hospital presentation, his wife noted that he had syncope and lost consciousness. As per collateral information from his brother and sister, who also noted the patient was shaking (tremulous) earlier that day:

“[He] was speaking nonsense, speaking a lot...hearing voices telling him that "he needed to die in order for others to live." He was talking about the world has already ended and they were only survivors.”

**Figure 1:**
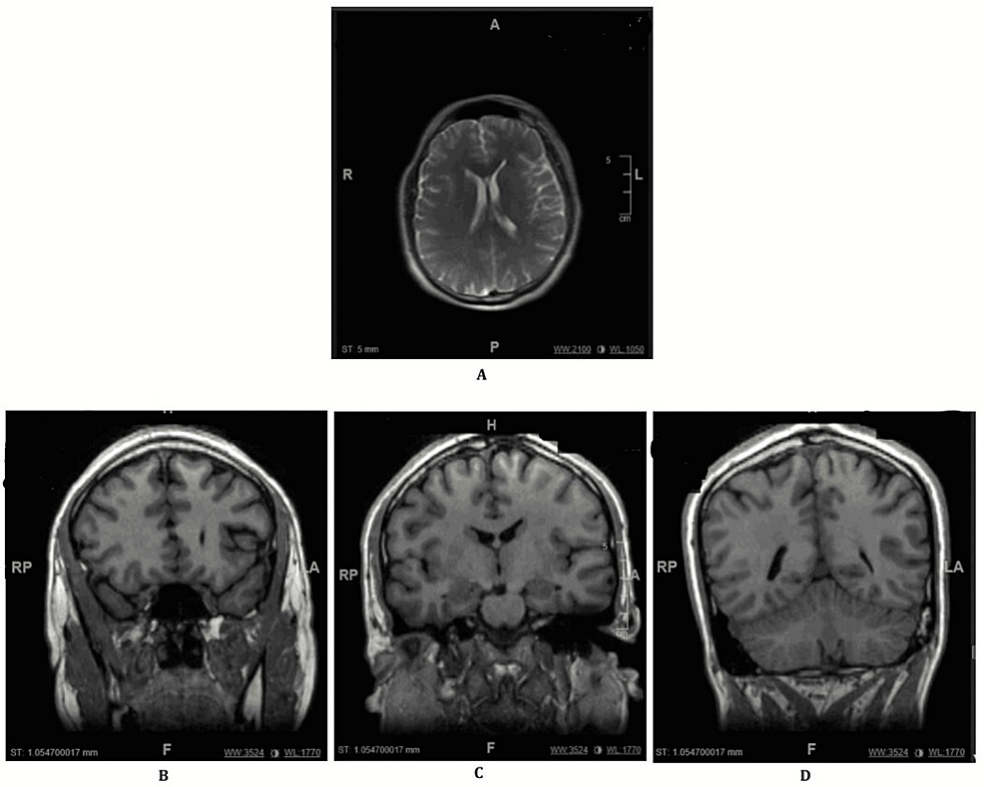
MRI images. Impression: No evidence of intracranial hemorrhage, mass, or infarct. No abnormal parenchymal or extra-axial enhancement. (A) Dorsal slice of the patient's brain. (B-D) From left to right, anterior to posterior coronal slice of the patient's brain.

In the triage area, the patient appeared confused, petrified of the floor, and was noted to be climbing up the door. Initial lab work revealed elevated white blood cell count (16.1), rhabdomyolysis (creatine phosphokinase level of 5,852 units/L), and high anion gap metabolic acidosis. Of note, the patient’s urine toxicology screen and blood alcohol levels were both negative, as mentioned above. In terms of imaging studies, the patient’s CT head without contrast, MRI of the brain without contrast, and Chest X-ray all demonstrated no acute pathology (Figure 1).

Once inside the psychiatric ER, he was found to be hypervigilant, paranoid (often scanning the room), and intermittently aggressive. For his own safety, as well as others’, he required multiple emergent intramuscular (IM) medications (Haldol 5 mg IM, Ativan 2 mg IM, and Benadryl 50 mg IM in combination) and 4 point restraints, though these had minimal effects on his behavior. Staff members found him attempting to disrobe during interviews with the treatment team, appearing confused, visually hallucinating, attempting to elope, and making bizarre statements, such as “There are snakes running around.” At this time, Neurology consultation was ordered and the impression was “post traumatic epilepsy,” with recommendations to obtain electroencephalography (EEG) in addition to MRI (no abnormal findings in either test). Due to the persistence of his rhabdomyolysis, low-grade fever of 100.5, and tachycardia (111 bpm), the patient was transferred to the medical ER for further evaluation.

The patient was subsequently admitted as an inpatient under the management of Family Medicine. Despite further restraints and IM medication, the patient remained combative and agitated. Neurology followed up and recommended lumbar puncture (LP). The patient’s cerebrospinal fluid (CSF) analysis initially revealed oligoclonal banding and a positive result for Coxsackie antibodies, prompting further testing to rule out secondary CNS infection and to explore autoimmune etiologies. The patient’s anti-NR1 antibody returned positive, confirming the diagnosis of anti-NMDA receptor encephalitis (Tables 1-3).

**Table 1:** CSF Findings WBC - white blood cells; RBC - red blood cells; CSF - cerebrospinal fluid; Ab(s) - antibody; CSF - cerebrospinal fluid; T. gondii - Toxoplasma gondii; VZV - varicella-zoster virus; Ds DNA - double-stranded deoxyribonucleic acid; HSV - herpes simplex virus; VDRL - Venereal Disease Research Laboratory test; SM - anti-Smith antibody; RNP - antinuclear ribonucleoprotein; ANA - antinuclear antibodies; IFA - immunofluorescence assay;  HIV - human immunodeficiency virus; Ab - antibody; Ag - antigen.

Component	Reference Range and Units	Result
Appearance	Clear	Clear
Color	Clear	Clear
Fluid type	0%-6%	CSF panic
Xanthochromia	Absent	Negative
WBC	0-5 mm^3^	153 High
RBC	No RBCs, mm^3^	2
Lymphs, CSF	40%- 80%	90 High
CSF mononuclear cells		10
Number of cells, CSF		Leave it blank panic
Glucose, CSF	40.0-70.0 mg/dL	75.3 High
Protein CSF	12.0-60.0 mg/dL	64.3 High
CSF chloride	120.0-130.0 mEq/L	123
Protein, total (CSF)	15-45 mg/dL	45
Pre-albumin (CSF)	1.3-6.9%	2.7
Albumin	51.9-67.8%	58.3
Alpha-1-globulin	1.8-6.5%	4.8
Alpha-2-Globulin	4.6-10.8%	4.3 Low
Beta globulin	7.8-18.2%	16.7
Gamma globulin	4.8-17.6%	13.2
Syphilis VDRL quantitation CSF	Nonreactive	Nonreactive
Xpert Xpress SARS-CoV-2	Negative	Negative
T. gondii IgG, CSF	<0.90 Antibody not detected; 0.90-1.09 equivocal; $\geq$1.10 antibody detected	<0.90
HSV 1 IgG Index	$\leq$1.00 Antibody not detected; >1.00 antibody detected	0.25
HSV 1 IgG Index	$\leq$1.00 Antibody not detected; >1.00 antibody detected	<0.01 VC
WNV IgG CSF	<1.30 index	<1.30
WNV IgM CSF	<0.90 index	<0.90 VC
Coxsackie B1 Ab, CSF	<1:1	1:1 High
Coxsackie B2 Ab, CSF	<1:2	1:1 High
Coxsackie B3 Ab, CSF	<1:3	<1:1
Coxsackie B4 Ab, CSF	<1:4	<1:1
Coxsackie B5 Ab, CSF	<1:5	1:2 High
Coxsackie B6 Ab, CSF	<1:6	1:2 High
Crypto Ag, CSF	Negative	Negative

**Table 2:** Blood Laboratory Findings WBC - white blood cells; RBC - red blood cells; CSF - cerebrospinal fluid; Ab(s) - antibody; CSF - cerebrospinal fluid; T. gondii - Toxoplasma gondii; VZV - varicella-zoster virus; Ds DNA - double-stranded deoxyribonucleic acid; HSV - herpes simplex virus; VDRL - Venereal Disease Research Laboratory test; SM - anti-Smith antibody; RNP - antinuclear ribonucleoprotein; ANA - antinuclear antibodies; IFA - immunofluorescence assay;  HIV - human immunodeficiency virus; Ab - antibody; Ag - antigen.

Component	Reference Range and Units	Result
NMDA Ab - Interpretation		Positive (comment: this test detected abnormal levels of anti-NR1 antibodies)
WBC	4.8-10.8 k/uL	6.2
RBC	4.50-5.90 M/uL	4.55
HGB	14.0-18.0 g/dL	13.6 Low
Hematocrit	42.0-52.0%	40.0 Low
Mean corpuscular volume	80.0-94.0 fL	87.9
Mean corpuscular hemoglobin	27.0-31.0 pg	30
Mean corpuscular hemoglobin concentration	32.0-36.0 g/dL	34.1
Red cell distribution width	11.5-14.5%	15.4 High
Mean platelet volume	7.2-10.4 fL	8.3
Platelets	130-400 k/uL	293
Neutrophils auto	44.0-80.0%	74.4
Lymphocytes auto	13.0-43.0%	14.6
Monocytes auto	2.0-15.0%	7.9
Eosinophils auto	0.0-3.0%	2.4
Basophils auto	0.0-3.0%	0.7
Neutrophils absolute	2.1-8.6 k/uL	4.6
Lymphocytes absolute	0.6-4.6 k/uL	0.9
Monocytes absolute	0.1-1.6 k/uL	0.5
Eosinophils absolute	0.0-0.9 k/uL	0.1
Basophils absolute	0.0-0.4 k/uL	0
NRBC instrument	None %/100 WBC	0.1
Nucleated RBC	None/100 WBC	0
Nucleated RBC absolute count	None k/uL	0.01
Hepatitis C Ab	Negative	Negative
Hepatitis B surface Ag	Negative	Negative
Sjogren's Abs (SS-A)	<1.0 NEG AI	<1.0
Sjogren's Abs (SS-B)	<1.0 NEG AI	<1.0
ds DNA Ab	≤4 IU/mL	<1
ANA screen, IFA	Negative	Negative VC
Quantiferon-TB plus	Negative	Negative
Nil	IU/mL	0.01
TB1-Nil	IU/mL	0.01
TB2-Nil	IU/mL	0.04
Mitogen-Nil IU/mL 7.70	IU/mL	7.70
HIV 1/2 AG/AB 4TH GEN	Nonreactive	Nonreactive
Procalcitonin	0.00-0.09 ng/mL	0.07
Sedimentation rate	0-15 mm/h	5
B-Hydroxybutyrate	0.02-0.27 mmol/L	0.27
Cholesterol	99-200 mg/dL	161
Triglycerides	40-150 mg/dL	91
HDL cholesterol	40-80 mg/dL	27 Low
LDL cholesterol calculated	60-130 mg/dL	116
Chol/HDL ratio		6
LDL/HDL ratio		4
Alcohol screen	0.0-10.0 mg/dL	<10.0
Treponema	Nonreactive	Nonreactive

**Table 3:** Urine Toxicology UR - urine; PCP - phencyclidine.

Component	Reference Range and Units	Result
UR amphetamines screen	Negative	Negative
Barbiturate screen, Ur	Negative	Negative
Benzodiazepine screen, urine	Negative	Negative
Cannabinoid screen, Ur	Negative	Negative
UR cocaine screen	Negative	Negative
UR methadone screen	Negative	Negative
Opiate screen, urine	Negative	Negative
PCP screen, Ur	Negative	Negative

At this time in his hospital course, Infectious Disease and Hematology/Oncology were also consulted for recommendations. A treatment regimen was agreed upon with Solu-Medrol pulse therapy (1 g daily for three days followed by maintenance dosing for 2-4 weeks) with intravenous immunoglobulin (IVIG) for augmentation. The psychiatric consultation team initiated the patient on Depakene 750 mg twice daily and Zyprexa 5 mg twice daily for the management of agitation and his aggressive behavior. The patient's main symptoms needing to be controlled, from a psychiatric perspective, were his level of aggression as well as psychotic features comprising auditory and visual hallucinations. It was thought that a second-generation atypical antipsychotic like Zyprexa would be effective in helping the latter, and that Depakote, as it often does in patients requiring mood stabilization, would help with the aggression the patient was displaying. At this point, though, it was clear that the only way to eliminate symptoms entirely would be to address the underlying cause, which was anti-NMDA receptor encephalitis, and so the psychotropics were thought of as a "bridge" to the treatment for the encephalitis. The final psychiatric conclusion was that these agents should be tapered off with more than two weeks of sustained/intact sensorium or at the discretion of another psychiatrist/neurologist. Clinically, the patient improved only mildly with pulse steroid therapy and IVIG. For example, mental status improved initially, but the patient again decompensated, demonstrating bizarre and severely agitated behavior, punching through a window on one occasion.

His complicated hospital stay necessitated extensive communication with all teams involved. The tentative Family Medicine disposition plan was to transfer the patient back to inpatient psychiatry, as the prevailing sentiment was that there was nothing further that could be done for the patient medically. After multiple discussions with Family Medicine, Oncology, Infectious Disease, and Neurology, a decision to initiate second-line therapy with cyclophosphamide was made. Initially, the patient’s family did not consent, opting to medicate him only if he became agitated or aggressive, and requested a transfer to another facility. Ultimately, the patient’s sister (his appointed healthcare proxy) felt he was making incremental improvements in cognitive functioning, and finally agreed to the cyclophosphamide treatment.

In the days following the initial dose, the patient was alert, oriented to person, place, and time, was able to have complete conversations with family members, and was noted with brighter affect (laughing and joking appropriately with staff and family). He became increasingly goal-oriented, stating that he wanted to return to work to support his family. After a total length of stay of 38 days, he was discharged, and outpatient reports show that he has been able to return to work and is actively engaged in outpatient treatment with his primary care physician and neurologist.

## Discussion

Our case review outlines the clinical scenario of a young male patient presenting with impaired orientation, bizarre behavior, and visual hallucinations. At first, he was treated with conventional psychotropic medications; however, there was no improvement noted in clinical status. A careful, systematic review of the clinical symptoms raised concerns about the possibility of anti-NMDA receptor encephalitis and was later confirmed with LP and CSF analysis.

The disease process is often first identified through clinical symptoms, and psychiatrists will invariably find themselves assessing such patients, as the prodromal period and initial symptomatology have significant overlap. The diagnostic criteria for anti-NMDA receptor encephalitis require rapid onset of fewer than three months of at least four of the six major symptom groups: abnormal behavior or cognitive dysfunction, speech dysfunction, movement disorders, dyskinesias, or rigidity/abnormal postures, and decreased level of consciousness, which were all present in the patient. The other criteria include seizures and autonomic dysfunction or central hypoventilation, which were inconclusive or uncertain by history. These criteria are only one piece of the three components to diagnose anti-NMDA receptor encephalitis, with the other two being abnormalities in CSF and the exclusion of other reasonable etiologies. The definitive diagnosis came from positive antibody testing [5]. The differential diagnosis of this clinical presentation includes primary psychiatric disorders (acute psychosis or schizophrenia), malignant catatonia, neuroleptic malignant syndrome, viral encephalitis, and encephalitis lethargica. EEG and brain MRI are generally performed to rule out other etiologies. Diagnosis is confirmed via CSF IgG antibody testing.

A dilemma exists regarding CSF versus serum, in the context of which is more authentic, and the sensitivity of antibody detection in CSF is much more clinically and statistically significant than in serum (sensitivity 100% [98.5-100%] versus 85.6% [80.7-89.4%], p<0.0001) [5]. Dalmau and colleagues endorse both [6-9]. Because of cost-effectiveness, clinicians may consider ordering antibody testing, where standard antipsychotic pharmacotherapy failed to improve the clinical display. Neuroimaging like MRI scanning is found within normal limits in 70% of cases [10]. Another test, an EEG, can help differentiate between encephalitis and primary psychiatric disorder by showing nonspecific slowing during the course of anti-NMDA receptor encephalitis (90%) [10]. Findings from PET scans have not demonstrated equivalent efficacy in establishing the diagnosis of anti-NMDA receptor encephalitis.

Treatment options include immunosuppression and tumor resection when indicated (the first line is generally pulsed IV steroids with the adjunct of IVIG if necessary). The treatment team considered therapeutic plasma exchange; however, upon reviewing a meta-analysis, it was noted that plasmapheresis was in fact an option, but due to the patient's presentation, and given that the literature shows no significant difference in efficacy, pulse steroids +/- IVIG was the more appropriate route for the patient [11]. Most patients will make a full recovery within two years of disease onset. Predictors of positive outcomes include the presence of a tumor, prompt diagnosis, and aggressive treatment including second-line therapies. There is relatively little prospective and randomized data, so treatment decisions should be individualized and take into consideration the patient's age, the presence or absence of a tumor, and symptom severity. Dalmau’s original case series demonstrates that about 75% of anti-NMDA receptor encephalitis patients recover to their neurological baseline or show only mild residual deficits, while the other remaining 25% may face dire complications or expire. Consecutive studies suggest a 12-24% chance of relapse [12-15].

## Conclusions

This case report presents the clinical vignette of a 30-year-old male patient, who presented with altered sensorium, disorganized behavior, and perceptual disturbance. Initially, he was put on standard psychotropic medications that did not help. After a careful review, the patient was diagnosed with anti-NMDA receptor encephalitis endorsed by the identification of CSF antibodies. There are several points to be highlighted within this case study. Initially, clinical suspicion and keeping a broad differential, despite what may appear as exclusively psychiatric symptoms, are important to prevent any delay in the appropriate testing or procedures that can hone in on the diagnosis. Successful treatment hinges on prompt induction of first-line treatment with steroids, and potentially second-line therapy with adjunct IVIG or immunosuppressants. Communication within the multidisciplinary approach, often combining clinicians from a myriad of subspecialties, is paramount to positive patient outcomes. Clinicians should remain alert and suspicious regarding the possibility of anti-NMDA receptor encephalitis in a patient with predominantly disorganized or psychotic symptoms who may not be responding to conventional antipsychotic therapy.
